# Confusion matrix and minimum cross-entropy metrics based motion recognition system in the classroom

**DOI:** 10.1038/s41598-022-07137-z

**Published:** 2022-02-23

**Authors:** Ming-Te Wu

**Affiliations:** Department of Information Technology, Kao Yuan University, No. 1821, Zhongshan Rd., Luzhu Dist., Kaohsiung, 82151 Taiwan

**Keywords:** Computer science, Information technology

## Abstract

This research proposes a motion recognition system for early detection of students' physical aggressive behavior in the classroom. The motion recognition system recognizes physical attacks so that teachers can resolve disputes early to reduce other greater injuries. In the beginning, cameras were used in this system to monitor students’ classroom activities and to obtain body images by removing background and saliency maps. Two angles from arm to shoulder and shoulder to the center of the body are then measured and the velocity between the two frames from the movement of the body is detected, and use these angle and velocity values as the criterion for judging whether it is a physical attack. In the end, the accuracy of the proposed algorithms is verified by using the confusion matrix based on machine learning and the minimum cross entropy based on neural networks. The simulation proves that the proposed algorithm can correctly detect the attack behavior of the collected videos.

## Introduction

The report by Casas^1^ pointed out that regardless of whether the child uses indirect or direct physical aggression, there is a significant correlation between the parent's parenting style, attachment relationship, and psychological control behavior. The findings emphasize the importance of indirect and physical aggression and parenting behaviors of young children, as well as the potential connection between the child’s gender and the mother and father who raises the child. Kupersmidt^2^ showed that in the field of early childhood education, teachers estimated that 10% of preschool children have daily bullying behaviors. Aggressive behaviors appear in early childhood^3^, and even these early forms of aggressive behavior will always exist and eventually become a social problem^4^. Scholars' research found that^5^ teachers assessing children's aggressive behavior at the age of 8 are related to maladjustment in school in the future and lead to long-term unemployment in adulthood. Whenever aggressive behavior occurs, people almost always have habitual gestures. If a system can be developed that can detect aggressive gestures as soon as possible, it can reduce the occurrence of injuries. Many articles on gesture research have been published recently^6–9^. Kim^6^ developed a system that can change the 3-D spatial coordinates of each joint to the 2-D angle representation of the corresponding arm, and display the specific time pattern of each dynamic gesture by using a discrete hidden Markov model. A hand-raising detection system initially obtains motion data through time difference and then uses a threshold to obtain the object of interest proposed by Yao^7^. To realize long-distance human–computer interaction, Kim^8^ and Lupinetti^10^ developed an arm gesture detection system. The system initially recognizes the user's face and arms through the background removal method and then determines which gesture is based on the position where the arm is raised. An arm pose segmentation method without monitoring was proposed by Simão^9^. This method is based on the use of thresholds, which can divide the input continuous flow of information, including unsegmented and unbounded, into dynamic and static segmented types. Although these methods can detect gestures, most of them use thresholds as a solution to detect gestures. The threshold used to detect gestures can usually get good results, but if it is applied to attacks between people, the accuracy is insufficient. Akl^11,12^ published a gesture detection technology that uses data obtained from a 3-axis accelerometer, including the training phase and the test phase^13^. A study uses the three gesture detection technologies of artificial neural network and dynamic time warp, hidden Markov model at the same time, and compares these three methods with the accelerometer in the mobile equipment, and then finds that the best approach is the dynamic time warping^13^. To model the image characteristics of the surface electromyography signal, Tsinganos^14^ applied the Hilbert space-filling curve and classified it through a convolutional neural network. In recent years, other related articles have been put forward one after another^15–18^. Besides, there are many relevant and interesting studies for reference^19–21^. To improve the accuracy of detecting gestures, the above methods use artificial neural networks and convolutional neural networks. These methods have indeed improved the recognition ability, but there is still room for improvement inaccuracy. Occasionally, conflicts between students on campus cause mutual injuries. This kind of scene happens every year. Therefore, this motivates us to develop a motion recognition system that can detect physical conflicts as early as possible and immediately eliminate them to reduce serious injuries. Since the matrix is a very effective and popular modeling tool in various applications, some articles^22–26^ have been proposed and applied in other fields in recent years.

A scheme for identifying aggressive behaviors is proposed in this research and teachers can be notified immediately. When there are dangerous aggressive behaviors among students, teachers can eliminate conflicts as soon as possible to prevent aggressive behaviors. This technique uses some cameras to observe students' behavior in class, then background elimination algorithms and saliency map technologies are used to capture the body’s position. Next, the angle between the line formed from the arm to the shoulder and the line formed from the shoulder to the center of the body, and the velocity of movement of the center of the body are calculated. Whether the recognized body movement is an aggressive behavior is determined by the angle and velocity values in this algorithm. In the end, confusion matrix and minimum cross-entropy are applied, based on machine learning and neural network, to verify the accuracy of the proposed algorithm.

The rest of this article is divided into the following. The background of well-known gesture detection and the proposed method is described in “Background” and “The proposed motion recognition system”. “Experimental results” shows the simulation results. The conclusion of this manuscript is in “Conclusions”.

## Background

In this section, a visual attention system, confusion matrix, and cross entropy minimization are introduced as follows. “Visual attention system” presents the visual attention system, “Confusion matrix” introduces the confusion matrix, and “Cross entropy minimization” statements the cross entropy minimization used in this work.

### Visual attention system

To capture the region of interest (ROI), time segmentation is often used in video processing^7^. The difference image is calculated by subtracting each pixel in the current frame and the previous frame, namely,

1 $$D\left(i,j\right)={F}_{t}\left(i,j\right)-{F}_{t-1}\left(i,j\right)$$

where *D*(*i*, *j*) represents the difference between the two images, and *F*_*t-1*_(*i*, *j*) and *F*_*t*_(*i*, *j*) represent the previous frame and the current frame, respectively. The background information is deleted after subtracting two consecutive frames. Image changes in nature are easily affected by time segmentation methods, and a common influencing factor is the non-static background environment. It is a very useful method to eliminate the non-static background environment by using a threshold to check. Yeh^15^ define the pixel values of the same offset in two consecutive frames as static pixels and expressed as

2 $$if \left|\left|D\left(i,j\right)\right|\right|<Threshold, \,then D\left(i,j\right) \epsilon S\left(i,j\right)$$

where *S* (*i*, *j*) represents a group of static pixels. To find the region of interest, a visual attention system was proposed by Chen^17^. In the beginning, color quantization is used in this visual attention system to smooth the color in the texture area. The saliency map is then generated by using the transform color space method. A content-based saliency map is finally formed to calculate the contrast value. In visual attention analysis, the advantage of a content-based saliency map is that it can provide texture, edge intensity, color, and contrast information. In addition, the saliency map scheme is used to extract the region of interest. To highlight the color of the region of interest and smooth texture region, initially, a frame is divided into 4 × 4 blocks using color quantization in this method to avoid fragile areas due to texture. The value in the RGB color space field is then converted to the value in the XYZ color space field. By using the color space conversion scheme^18^, the expression is

3 $$\left[\begin{array}{l}X\\ Y\\ Z\end{array}\right]=\left[\begin{array}{ccc}0.412& 0.357& 0.18\\ 0.212& 0.715& 0.072\\ 0.019& 0.119& 0.95\end{array}\right]\left[\begin{array}{c}R\\ G\\ B\end{array}\right].$$

Next, the conversion from XYZ color space to LUV color space continues in the saliency map method, and the transformation r»ows.

4 $$L=\left\{\begin{array}{l}116\sqrt[3]{{y}_{r}}-16 {y}_{r}>\varepsilon \\ k{y}_{r} { y}_{r}\le \varepsilon \end{array}\right.$$

5 $$u=13L({u}^{^{\prime}}-{u}_{r}^{^{\prime}})$$

6 $$v=13L({v}^{^{\prime}}-{v}_{r}^{^{\prime}})$$

where $${y}_{r}=\frac{Y}{{Y}_{r}}$$, $$\varepsilon =0.008856$$, $$k=903.3$$,

7 $${u}^{^{\prime}}=\frac{4X}{X+15Y+3Z}$$

8 $${v}^{^{\prime}}=\frac{9\mathrm{Y}}{\mathrm{X}+15\mathrm{Y}+3\mathrm{Z}}$$

9 $${u}_{r}^{^{\prime}}=\frac{4{X}_{r}}{{X}_{r}+15{Y}_{r}+3{Z}_{r}}$$

10 $${v}_{r}^{^{\prime}}=\frac{9{Y}_{r}}{{X}_{r}+15{Y}_{r}+3{Z}_{r}}$$

In the end, the contrast values are computed by the obtained frame value from the color quantization and color space conversion. Assuming that there is one pixel for each perceptual unit, then an *N* × *M* size frame has *N* × *M* perceptual units. By the following equation, in other words, the contrast value ${C}_{i,j}$ is calculated.

11 $${C}_{i,j}=\sum_{q\epsilon A}e({p}_{i,j},q)$$

where *q* represents the perception unit and *p*_*i,j*_(*i* ∈ [0,*N*], *j* ∈ [0,*M*]). The term *e* is expressed as the Euclidean distance between *p*_*i*,*j,*_ and *q*, and A represents the area surrounding the perceived position (*i*, *j*). The area of A can be determined by adjusting the sensitivity of the perception field. By reducing the size of A, the perceptual field can be made more sensitive. For normalization, the contrast value *C*_*i*,*j*_ is set between 0 and 255 in this article. As long as the contrast value in the saliency map is used as the density, the center point in the saliency map can be regarded as the center of visual attention. The center of visual attention is therefore calculated

12 $$\left\{\begin{array}{c}{x}_{c}=\frac{1}{{C}_{T}}\sum_{j=0}^{N-1}{C}_{i,j}\times i\\ {y}_{c}=\frac{1}{{C}_{T}}\sum_{i=0}^{M-1}{C}_{i,j}\times j\end{array}\right.$$

where $${C}_{T}=\sum_{i=0}^{M-1}{\sum }_{j=0}^{N-1}{C}_{i,j}$$.

### Confusion matrix

Fawcett^19^, Powers^20^, and Stehman^21^ used the confusion matrix method to improve the accuracy of judging whether it is an attack. The confusion matrix can be illustrated in the table below.

**Table:** 

Video	Total frames	True condition			
		Positive	Negative		
Predicted outcome	Positive	True positive (TP)	False positive (FP)	Positive predictive value (PPV) $\frac{TP}{TP+FP}$	False discovery rate (FDR) $\frac{FP}{TP+FP}$
	Negative	False negative (FN)	True negative (TN)	False omission Rate (FOR) $\frac{FN}{FN+TN}$	Negative predictive value (NPV) $\frac{TN}{FN+TN}$
		True positive rate (TPR) Sensitivity $\frac{TP}{TP+FN}$	False positive rate (FPR) $\frac{FP}{FP+TN}$		
	Accuracy $\frac{TP+TN}{T}$	False negative rate (FNR) $\frac{FN}{TP+FN}$	True negative rate (TNR) $\frac{TN}{FP+TN}$		

TP (True Positive) indicates that "predicted outcome is positive" is the same as "true condition is positive." FP (False Positive) means that "the predicted outcome is negative" is different from "true condition is positive." TN (True Negative) means that "predicted outcome is negative" is the same as "true condition is negative." FN (False negative) means that "predicted outcome is negative" is different from "true condition is positive." T (Total Population) represents the number of predictions for all frames. Therefore, the accuracy can be calculated

13 $$\mathrm{A}=\frac{TP+TN}{T}$$

### Cross entropy minimization

There is an important function in the neural network learning process that can affect the quality of the model, called the loss function. The focus of the loss function is to calculate the gap between the output value and the actual value; when the gap between the output value and the actual value is larger, the value of the loss function is larger, and vice versa; therefore, the neural network has an important point in the learning process The purpose of is to minimize the loss function to achieve better classification or prediction results.

The cross entropy function is mainly to evaluate whether the output value and the actual value are very different. Cross entropy is a loss function for probability. Because it can effectively quantify the difference between predicted probability and actual probability, it is often used in classification problems. The formula is as follows,

14 $$-{\sum }_{i}{Y}_{i}\mathrm{ln}({y}_{i})$$

Among them, ${y}_{i}$ is the value of probability, and ${Y}_{i}$ is the probability of the actual category.

The proposed system with motion recognition ability was introduced below. The proposed system initially removes the background of the image and applies a saliency map scheme to extract the ROI part of the body. The proposed algorithms then use the movement speed of the body and the angle between the detected arm and the body in two consecutive frames to determine whether the detected motion is aggressive. Finally, this research exploits the confusion matrix and the minimized cross-entropy from the neural network-like basis, which is detailed in the next section.

## The proposed motion recognition system

**Figure 1:**
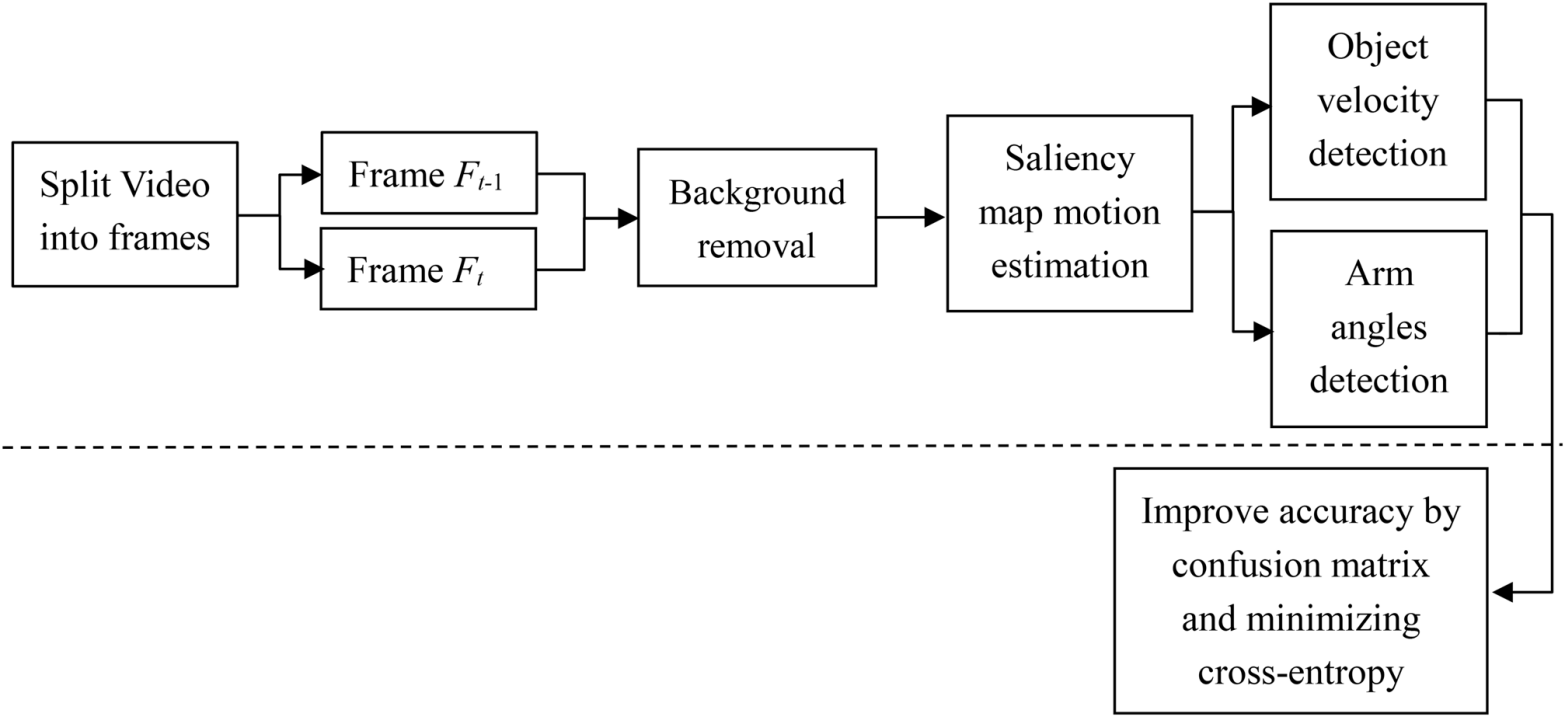
A general overview of the proposed motion recognition system.

Figure 1 shows a block diagram of the proposed system with motion recognition. Two stages are provided in the overview: the first stage is the block diagram above the dotted line, which is also the testing phase; the second stage is the block diagram below the dotted line, which is the training phase. In addition, five parts are included in the testing stage. Framing the captured video is the main work of the first part. Second, the comparison of two consecutive frames is performed in the second part. The removal of background and uninteresting background is in the third part. To get the desired ROI, the saliency map motion estimation is implemented in the fourth part. The last part detects the angle of the arm by the angle between the arm to the shoulder and the shoulder to the center of the body, as well as the velocity of the ROI. The training phase contains only one part, that is, by minimizing the cross-entropy and confusion matrix to compute the accuracy of the offensive behavior obtained by the candidate in the testing phase. To make this motion recognition system have the purpose of self-upgrading, the method of improving the accuracy in this paper is to minimize the cross-entropy and adjust the threshold of the ROI movement velocity. The detailed statement is as follows.

The position of the four cameras in the left, right, front, and rear of the classroom is intended to prevent the possibility of gestures being blocked by objects or people. Initially, the background removal is performed on the camera frame using Eqs. (1) and (2), and then the frame is color quantized, and then Eqs. (3) and (4) are used to calculate the color space transformation and contrast values, respectively. Therefore, a saliency map is acquired.

**Figure 2:**
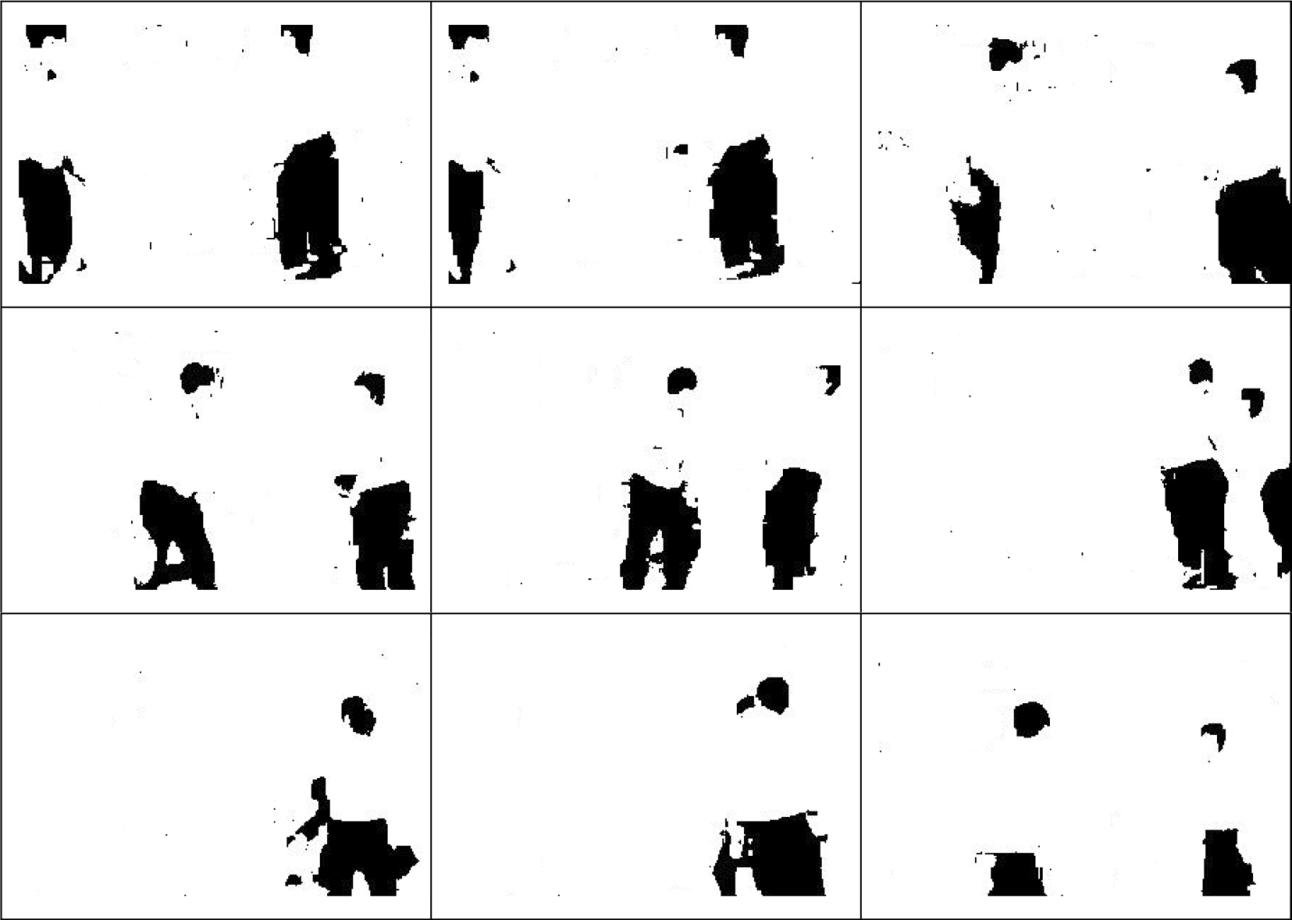
The ROI objects of the first nine frames.

Through Eq. (12), the center point of the ROI object is calculated, and the center position of the ROI in each frame is recorded at the same time after the saliency map is obtained. The center point coordinates of the ROI object, taking the first frame of Fig. 2 as an example, can be calculated as (5,4) and (16,4) by Eq. (12). The ROI object of two consecutive frames is used and the motion velocity of the object is calculated to determine whether the candidate's aggressive behavior is about to occur. Judging that an attack is about to happen is if the movement speed exceeds the threshold. Assuming that the center coordinates of the previous frame *F*_*t*−1_ and the current frame *F*_*t*_ are (*x*_*t*−1_, *y*_*t*−1_) and (*x*_*t*_, *y*_*t*_) respectively, then the velocity *V*_*t*−1_ = (*x*_*t*_* − x*_*t*−1_, *y*_*t*_* − y*_*t*−1_). Whether the candidate's behavior is offensive is determined by whether the velocity *V*_*t*−1_ of the current frame and the velocity *V*_*t*_ of the next frame is greater than the threshold. The two successive velocities of the object are ${V}_{t-1}=({x}_{t}-{x}_{t-1},{y}_{t}-{y}_{t-1})$ and ${V}_{t}=({x}_{t+1}-{x}_{t},{y}_{t+1}-{y}_{t})$ in consecutive pictures, namely, they must be greater than the threshold before they can be defined as an attack.

The total velocity of all objects must be summed if there are a total of n objects in a frame, and the following results are judged as candidate attack behaviors, namely,

15 $$\sum_{i=1}^{n}{V}_{t-1}>{T}_{t-1} \, and \, \sum_{i=1}^{n}{V}_{t}>{T}_{t},$$

where *T*_*t*−1_ and *T*_*t*_ are thresholds.

**Figure 3:**
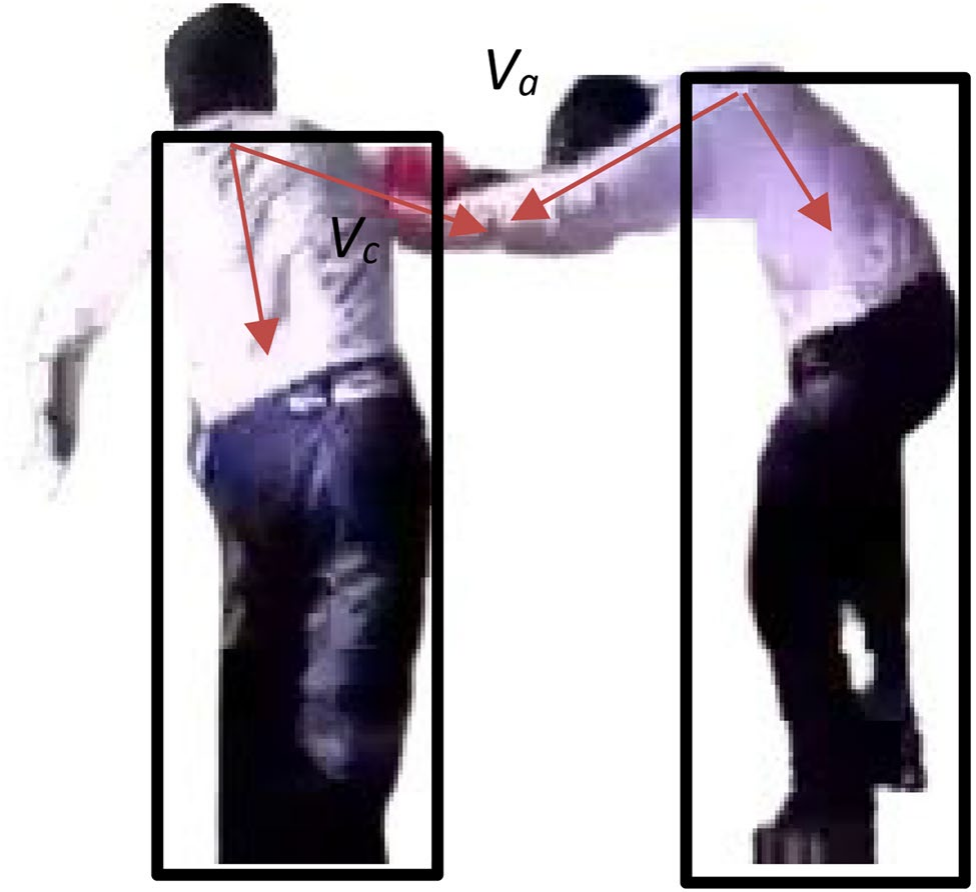
The vector *Va* is from the arm to the neck and the vector *Vc* between the center of the body and the neck.

**Figure 4:**
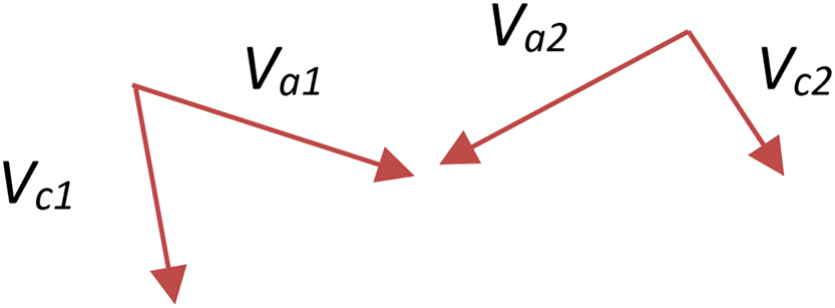
The angle between vector *Va* and *Vc*.

In this study, a rectangular line was used to extract the body contour of the ROI after removing the background. The center point of the neck was set as the center point of the upper edge of the body contour. When it exceeds the outline range, it will be deleted, and then only the outline of the head and arms can be kept. As shown in Fig. 3, the vector *V*_*a*_ from the arm to the neck then can be obtained by calculating the center point of the arm contour and the vector *V*_*c*_ can be obtained by calculating the distance between the center of the body and the neck. Next, as shown in Fig. 4, the angle between the two vectors can be obtained as $\theta ={\mathrm{tan}}^{-1}\frac{{V}_{a}}{{V}_{c}}$. The detected behavior is regarded as an attack if the angle $\theta$ is greater than the threshold *T*_*θ*_ as shown in the following equation.

16 $$\theta =\sum_{i=1}^{n}{\mathrm{tan}}^{-1}\frac{{V}_{ai}}{{V}_{ci}}>{T}_{\theta }$$

where *i* is the number of ROI. In the end, the calculation of the minimized loss function uses cross-entropy in this paper.

17 $$L=-{\sum }_{i}\left({TPR}_{i}+{FPR}_{i}\right)+\mathrm{ln}({PPV}_{i}+{NPV}_{i})$$

The optimal value is obtained by minimizing cross-entropy.

**Figure 5:**
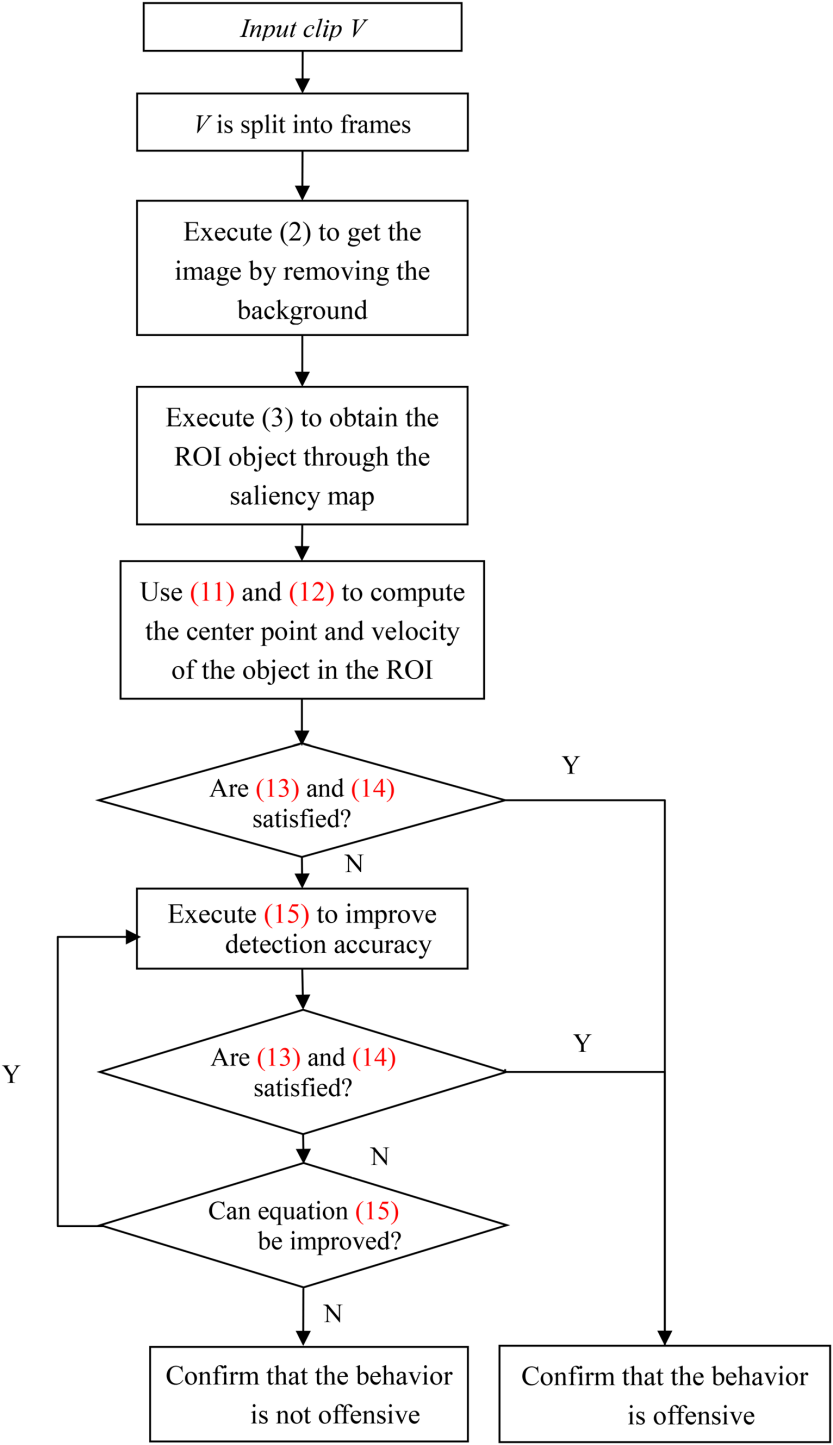
The flowchart of the proposed algorithms.

The thresholds *T*_*t*−1_ and *T*_*t*_ are then adaptively adjusted. The thresholds *T*_*t*-1_ and *T*_*t*_ are always adjusted if the accuracy obtained can be improved unless the accuracy value cannot be improved. Note that the accuracy is effectively continuously improved by adaptive adjustment of the thresholds of *T*_*t*−1_ and *T*_*t*_. In other words, Eq. (15) only detects candidate attack behaviors, while Eq. (13) records the accuracy of the adaptively adjusted thresholds *T*_*t*−1_ and *T*_*t*_. In addition, other data can be calculated, including false discovery rate (FDR), positive predictive value (PRV), predictive value (NPV), false missing rate (FOR), false-positive rate (FPR), negative true positive rate (TPR), true negative rate (TNR) and false-negative rate (FNR). These data can be passed through the following algorithms $\frac{FP}{TP+FP}$, $\frac{TP}{TP+FP}$, $\frac{TN}{FN+TN}$, $\frac{FN}{FN+TN}$, $\frac{FP}{FP+FN}$, $\frac{TP}{TP+FN}$, $\frac{TN}{FP+TN}$ and $\frac{FN}{TP+FN}$ are calculated, respectively. In these data, the most significant are the values of NPV and PRV. This is because NPV represents non-aggressive behavior and the prediction is also non-aggressive behavior, and PRV represents aggressive behavior and the prediction is also aggressive behavior. As shown in the flowchart in Fig. 5. The following steps are from the proposed system to determine if an attack has occurred.

1. For input clip *V*, *V* is split into frames.
2. Remove the background by executing Eq. (2).
3. Obtain the ROI object using the saliency map and execute Eq. (12).
4. Execute Eqs. (15) and (16) to calculate the velocity and angle of the ROI object. If Eqs. (15) and (16) are true, go to step 6. If not, go to the next step.
5. Execute Eq. (17) to improve detection accuracy. If Eq. (17) continues to improve, the values of *T*_*t*−1_ and *T*_*t*_ in Eq.  (15) and *T*_*θ*_ in Eq. (16) are adaptively adjusted. In another way, go to step 7.
6. Confirm that the behavior is offensive.
7. Confirm that the behavior is not offensive.

## Experimental results

The algorithm proposed in this section performs simulations on several collected video sequences to evaluate the accuracy of judging aggressive behaviors, such as " *Korea-students*", " *US-students*", " *Taiwan-students*-*Part I*" and " *Taiwan-students*-*Part II*", their frame numbers are 50, 45, 50 and 50, respectively. A confusion matrix is used as an objective measure of accurate quality. Tables 1, 2, 3 and 4 are the comparison between the predicted results and the real conditions in the test clips of the proposed method. The proposed algorithm shows excellent accuracy values as can be seen from Tables 1, 2, 3 and 4. It can be seen that this method has obtained very good results on Accuracy. For *Korea-students*, *US-students*, *Taiwan-students*-*Part I,* and *Taiwan-students*-*Part II* films, it can be increased to 0.96, 0.98, 1, and 1, respectively. For the " *US-students*" and " *Taiwan-students*" sequences, the best accuracy of the proposed algorithm is 0.98 and the total average accuracy of all videos obtained by the proposed algorithm is 0.975. To further understand, compared with the best accuracy of the " *US-students*" and "*Taiwan-students*" sequences, the proposed algorithm reduces the minimum accuracy of the "*Korea-students*" sequence by only 0.04. In short, the proposed method has good performance and In short, the proposed method has good performance and only slightly reduces the performance for the "*Korea-students*" sequence. The comparison of NPV and PPV regarding the real conditions and prediction results of different videos in the proposed scheme is shown in Table 5, where the terms NPV and PPV represent the negative predictive value and the positive predictive value, respectively. As the terms PRV and NPV of the estimation accuracy of aggressive and non-aggressive behaviors, the proposed algorithm has the highest estimation accuracy in the sequence of "*US-students*" and "*Taiwan-students*", which can be seen from Table 5. The NPV value of the "*US-students*" sequence is as high as 0.86 even though it is lower than other clips.

**Table 1:** Comparison of the predicted results and real conditions of the proposed scheme with the test fragments of *Korea-students*.

*Korea-students*	Total frames = 50	True condition	
		Aggressive	Non-aggressive
Predicted condition	Aggressive	22	2
	Non-aggressive	2	24
Accuracy		0.96

**Table 2:** Comparison of the predicted results and real conditions of the proposed scheme with the test fragments of *US-students*.

*US-students*	Total frames = 45	True condition	
		Aggressive	Non-aggressive
Predicted condition	Aggressive	38	0
	Non-aggressive	1	6
Accuracy		0.98

**Table 3:** Comparison of the predicted results and real conditions of the proposed scheme with the test fragments of *Taiwan-students-Part I*.

*Taiwan-students-Part I*	Total frames = 50	True condition	
		Aggressive	Non-aggressive
Predicted condition	Aggressive	38	1
	Non-aggressive	0	11
Accuracy		0.98

**Table 4:** Comparison of the predicted results and real conditions of the proposed scheme with the test fragments of *Taiwan-students-Part II*.

*Taiwan-students-Part II*	Total frames = 50	True condition	
		Aggressive	Non-aggressive
Predicted condition	Aggressive	19	1
	Non-aggressive	0	30
Accuracy		0.98

**Table 5:** The prediction results and real conditions of the proposed scheme with different videos are compared concerning PPV and NPV.

	Video	True condition	
		PPV	NPV
Predicted condition	*Korea-students*	0.92	0.92
	*US-students*	1	0.86
	*Taiwan-students-Part I*	0.97	1
	*Taiwan-students-Part II*	0.95	1

It can be seen from Table 6 that our proposed method proves that the objective performance evaluation in terms of accuracy is superior to Patwardhan^27^, Veenendaal^28^, and Goyal's^29^ scheme, the accuracy is about 0.02–0.19. The proposed method outperforms the schemes of Patwardhan, Veenendaal, and Goyal with an accuracy rate of about 0.02–0.18 dB in the *Korea-students* sequence. The proposed method outperforms the schemes of Patwardhan, Veenendaal, and Goyal with an accuracy of about 0.02–0.17 dB in the same comparison in the *US-students* sequence. The proposed method is better than the schemes of Patwardhan, Veenendaal, and Goyal. The accuracy rate of about 0.03–0.19 dB is also being compared in the sequence of Taiwanese students. The proposed method is better than the schemes of Patwardhan, Veenendaal, and Goyal with an accuracy rate of about 0.03–0.19 dB, and is also being compared in the sequence of *Taiwan-students*.

**Table 6:** Compare the proposed scheme with Patwardhan, Veenendaal, and Goyal using accuracy videos for *Korean-students*, *US-students*, and *Taiwan-students*.

Video	Accuracy			
	Patwardhan	Veenendaal	Goyal	Proposed
*Korea-students*	0.91	0.78	0.94	0.96
*US-students*	0.93	0.81	0.96	0.98
*Taiwan-students-Part I*	0.92	0.79	0.95	0.98
*Taiwan-students-Part II*	0.94	0.82	0.96	0.98

## Conclusions

In this study, a motion recognition system using saliency map technology and background removal is proposed, and the accuracy is improved by confusion matrix and minimized cross-entropy. The ROI object of the frame is obtained by the method of saliency map and background removal. Whether it is an aggressive behavior is determined by detecting the angle between the velocity of the arm relative to the neck and the velocity of the center of the body relative to the neck. The accuracy of the proposed algorithm is improved by implementing a method based on a confusion matrix and minimizing cross-entropy. The attack behavior of the collected clips can be accurately detected and verified by the system based on the experimental results. It is proved by simulation that excellent accuracy performance can be achieved, such as several fragments of *Korea-students*, *US-students*, *Taiwan-students-Part I,* and *Taiwan-students-Part II*.
